# Effects of remote ischemic preconditioning in hepatectomy: a systematic review and meta-analysis

**DOI:** 10.1186/s12871-024-02506-9

**Published:** 2024-03-26

**Authors:** Chun Tian, Aihua Wang, He Huang, Youwan Chen

**Affiliations:** 1 Department of Anesthesiology, Yongchuan Hospital of Chongqing Medical University, Chongqing, 402160 China; 2 Department of Critical Care Medicine, Chongqing Yongchuan District People’s Hospital, Chongqing, 402160 China; 3 Department of Anesthesiology, The Second Affiliated Hospital of Chongqing Medical University, Chongqing, 400010 China

**Keywords:** Remote ischemic preconditioning, Ischemia-reperfusion injury, Hepatic ischemia-reperfusion injury, Hepatectomy, Liver resection

## Abstract

**Background:**

Animal experiments have confirmed that remote ischemic preconditioning (RIPC) can reduce hepatic ischemia-reperfusion injuries (HIRIs), significantly improving early tissue perfusion and oxygenation of the residual liver after resections, accelerating surgical prognoses, and improving survival rates. However, there is still controversy over the role of RIPC in relieving HIRI in clinical studies, which warrants clarification. This study aimed to evaluate the beneficial effects and applicability of RIPC in hepatectomy and to provide evidence-based information for clinical decision-making.

**Methods:**

Randomized controlled trials (RCTs) evaluating the efficacy and safety of RIPC interventions were collected, comparing RIPC to no preconditioning in patients undergoing hepatectomies. This search spanned from database inception to January 2024. Data were extracted independently by two researchers according to the PRISMA guidelines. The primary outcomes assessed were postoperative alanine transaminase (ALT), aspartate transaminase (AST), total bilirubin (TBIL), and albumin (ALB) levels. The secondary outcomes assessed included duration of surgery and Pringle, length of postoperative hospital stay, intraoperative blood loss and transfusion, indocyanine green (ICG) clearance, hepatocyte apoptosis index, postoperative complications, and others.

**Results:**

Ten RCTs were included in this meta-analysis, with a total of 865 patients (428 in the RIPC group and 437 in the control group). ALT levels in the RIPC group were lower than those in the control group on postoperative day (POD) 1 (WMD = − 59.24, 95% CI: − 115.04 to − 3.45; *P* = 0.04) and POD 3 (WMD = − 27.47, 95% CI: − 52.26 to − 2.68; *P* = 0.03). However, heterogeneities were significant (*I*^*2*^ = 89% and *I*^*2*^ = 78%), and ALT levels on POD 3 were unstable based on a sensitivity analysis. AST levels on POD 1 in the RIPC group were lower than those in the control group (WMD = − 50.03, 95% CI: - 94.35 to − 5.71; *P* = 0.03), but heterogeneity was also significant (*I*^*2*^ = 81%). A subgroup analysis showed no significant differences in ALT and AST levels on POD 1 between groups, regardless of whether the Pringle maneuver or propofol was used for anesthesia (induction only or induction and maintenance, *P* > 0.05). The remaining outcome indicators were not statistically significant or could not be analyzed due to lack of sufficient data.

**Conclusion:**

RIPC has some short-term liver protective effects on HIRIs during hepatectomies. However, there is still insufficient evidence to encourage its routine use to improve clinical outcomes.

**Trial registration:**

The protocol of this study was registered with PROSPERO (CRD42022333383).

**Supplementary Information:**

The online version contains supplementary material available at 10.1186/s12871-024-02506-9.

## Introduction

Hepatectomy is a basic and effective treatment for primary and secondary liver malignancies that improves survival rates, particularly for patients with early and middle stage localized disease [1, 2]. With the development of modern medicine, precise hepatic segmentectomies are becoming increasingly mature, and as such higher requirements are needed for anesthesia and perioperative management [3]. The main surgical problem during segmental hepatectomies is intraoperative blood loss. During liver resections, intermittent portal vein triple clamping (Pringle maneuver) is associated with controlled low central venous pressure, which reduces intraoperative blood loss [2, 4]. However, subsequent tissue ischemia and reperfusion may lead to hepatic ischemia-reperfusion injuries (HIRIs), which usually occur when blood supply to the liver is temporarily blocked and subsequently restored [5].

The mechanisms involved in HIRIs are complex and yet to be fully understood. These include the adhesion of white blood cells to endothelial cells, the activation of Kupffer cells, the release of inflammatory cytokines, free radicals, nitric oxide and adenosine, the induction of the inflammatory cascade, and cellular apoptosis [6, 7]. Tolerance of liver tissue to ischemia depends on several factors, such as duration of ischemia, liver collateral circulation, and liver metabolic needs, among others. Therefore, it is difficult to determine the exact safe ischemic time for each surgery. On the other hand, while the restoration of blood flow is essential to prevent irreversible liver cell damage, the reperfusion itself may aggravate ischemic liver cell damage.

After an extensive hepatectomy, HIRI of the residual liver may be a serious complication, leading to postoperative liver dysfunction and increased mortality [5]. In order to protect the residual liver from HIRI, several techniques have been used, including drugs and ischemic preconditioning, or remote ischemic preconditioning (RIPC), none of which are established as standard of care. Organ protection by RIPC began with the study of cardiac muscle protection, which involves repeated temporary cessation of blood flow to the limbs [5, 8]. RIPC procedures are non-invasive and therefore a more suitable method to reduce HIRI.

Even though RIPC has been shown to have hepatoprotective effects in several animal experiments [9–11], patient-based studies have shown controversial results [12–14]. Only two systematic reviews on this topic were found in the literature [15, 16], both of which contained fewer studies and less data than this study. In addition, one mistakenly included patients with remote ischemic postconditioning (RIpostC) in the meta-analysis, and the other included a randomized controlled trial (RCT) of liver transplant recipients. Moreover, these were published within a year of each other, despite yielding conflicting conclusions. Hence, this study aimed to provide an updated systematic review of the perioperative effects of RIPC in patients undergoing hepatectomy. The study’s hypothesis is that RIPC is beneficial in reducing the effects of HIRI in patients undergoing hepatectomy.

### Methods

This systematic review was prepared in concordance with PRISMA and AMSTAR2 recommendations to assess methodological quality [17, 18]. RCTs were included to compare perioperative outcomes in patients undergoing hepatectomy with or without RIPC. This systematic review has been registered with PROSPERO, under registration number CRD42022333383.

### Search strategy

Articles published until December 2023 were searched via PubMed, OVID, Web of Science, Cochrane library clinical trial databases, Embase, and other sources without language restrictions. Search terms consisted of various combinations of ‘remote ischemic preconditioning’, ‘distant ischemic preconditioning’, ‘remote ischemic conditioning’, ‘remote ischemic adaptation’, ‘limb ischemic preconditioning’, or ‘RIPC’, and ‘hepatectomy’, ‘liver resection’, ‘liver transplantation’, ‘liver graft’, or ‘hepatic ischemia-reperfusion’. In addition, references of included studies and other existing meta-analyses were collected to obtain additional eligible studies (Supplementary Table S1).

### Inclusion and exclusion criteria

Four researchers independently reviewed and retrieved all full-text articles simultaneously. Different views were discussed among the four researchers, and duplicated articles in databases were merged. When duplicate studies were found from the same population, the latest or most complete study was included.

The inclusion criteria were as follows: (1) subjects were patients undergoing hepatectomy or living donor hepatectomy; (2) intervention was RIPC versus control group without RIPC; (3) research type was prospective RCT; (4) outcomes were postoperative liver synthetic function.

The exclusion criteria were as follows: (1) animal experimental studies and ex vivo, in vitro or in silico model studies; (2) retrospective or single-arm studies; (3) case studies, cross-over studies, studies without a separate control group, editorials, meta-analyses and reviews; (4) abstract only studies; (5) studies without postoperative aminotransferase levels or data from review articles.

### Data extraction

Two researchers (Chun Tian and Aihua Wang) independently extracted data from each article. Any disagreements were resolved by consensus of a third researcher (He Huang). The following information was extracted from the included articles: the first author, year of publication; country or region of study, type of study, sample size, demographic data, outcomes, among others. If this information was not available in the study’s text, study graphs were enlarged and measured using the Grab software. In instances where data were not reported or unclear, researchers were contacted via e-mail (max. 2 attempts).

### Assessment of methodological quality and risk of bias

Included RCTs were assessed using the recommended Cochrane Collaboration biased-risk assessment table. This assessment was carried out independently by four researchers. Any disagreements were resolved by consensus. The biased-risk assessment table included random sequence generation, allocation concealment, blinding of participants and personnel, blinding of outcome assessment, incomplete outcome data, selective reporting, and other bias. Each study was classified as high, low, or uncertain risk.

The methodological quality of the results was evaluated using the Grades of Recommendation, Assessment, Development, and Evaluation (GRADE) guidelines. Ultimately, the quality of evidence for each outcome was rated as high, moderate, low, or very low.

### Primary and secondary outcomes

The primary outcomes evaluated in this study were those directly related to postoperative liver synthetic function, including postoperative alanine transaminase (ALT), aspartate transaminase (AST), total bilirubin (TBIL), and albumin (ALB) levels. The secondary outcomes assessed included duration of surgery and Pringle, length of postoperative hospital stay, intraoperative blood loss and transfusion, indocyanine green (ICG) clearance, hepatocyte apoptosis index, postoperative complications, and others.

### Statistical analysis

Statistical analysis was performed using the Review Manager 5.4 software. Continuous outcomes were reported as weight mean differences (WMD) with 95% confidence intervals (CI), and dichotomous outcomes were presented as odds ratios (OR) with 95% CI. In order to quantify inconsistencies of studies included in the meta-analysis, Cochran’s Q-test and *I*^*2*^ statistics were used. Low heterogeneity was considered when *I*^*2*^ ≤ 50%, and the fixed-effect model was used for analyses. Moderate heterogeneity was considered when *I*^*2*^ > 50% and high heterogeneity when *I*^*2*^ > 75%, and the random-effects model was used for analyses. Subgroups analyses or sensitivity analyses were then performed, and a descriptive analysis was conducted if a meta-analysis was inappropriate. Publication bias was assessed using funnel plots. Results were considered statistically significant when *P* < 0.05.

## Results

### Study characteristics

**Fig. 1:**
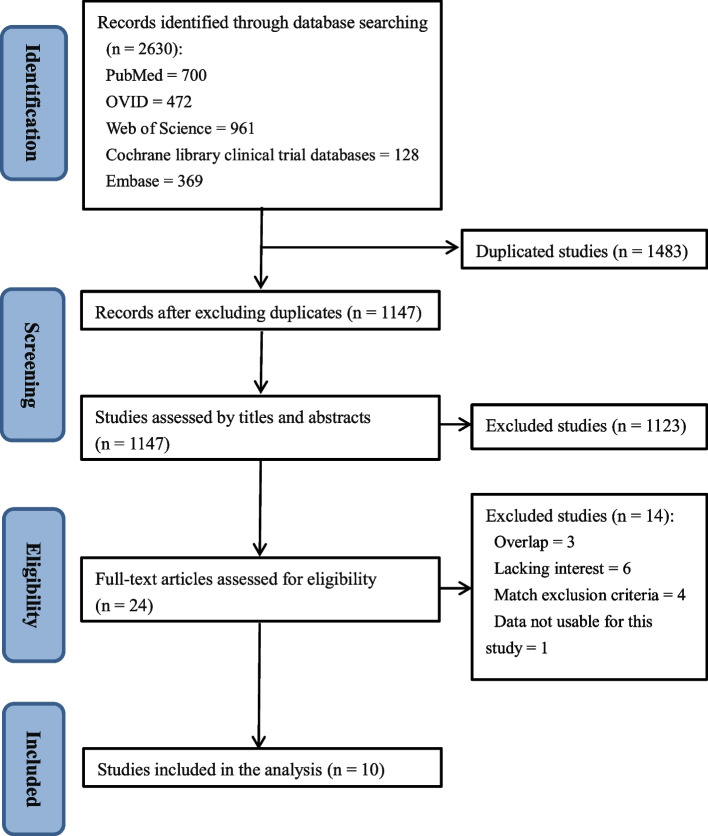
Flowchart of preferred reporting items for systematic reviews and meta-analyses (PRISMA) method for article selection

A total of 2630 relevant articles were initially identified, of which 1483 were duplicates. Excluding duplicates, a total of 1147 studies remained. After analyzing article titles and abstracts, 1123 articles did not meet the study criteria and were also excluded, leaving 24 studies for full-text review. After review, 10 RCTs [12–14, 19–25] met the eligibility criteria for data synthesis (Fig. 1).

The 10 prospective RCTs included a total of 865 patients undergoing hepatectomy (428 in the RIPC group and 437 in the control group). All included studies evaluated the liver synthetic function of postoperative residual livers using transaminase or TBIL levels. The basic characteristics of these studies are summarized in Table 1.

**Table 1:** Characteristics of the included studies

Author	Year	Country	Number		Age (years)		Gender (M / F)		Preconditioning (Limb, cycle, pressure)	Primary outcomes
			RIPC	Control	RIPC	Control	RIPC	Control		
Kanoria [25]	2017	UK	8	8	58–77	66–74	7/1	6/2	Right upper thigh, 3 × 10 /10 min, twice the measured systolic arterial pressure	ALT and AST levers on POD 1, and ICG clearance at immediately post-resection
Rakić [14]	2018	Croatia	20	20	63.8 (48–79)		59.4%/40.6%		Right upper limb, 3 × 5 /5 min, 200 mmHg	Liver synthetic function
Liu [24]	2019	China	69	67	51.7 ± 10.6	52.1 ± 10.9	59/10	59/8	Right arm, 3 × 5 /5 min, 225 mmHg	Postoperative peak level of TBIL
Jung [13]	2020	Korea	75	73	29 (24, 35)	28 (25, 35)	54/21	51/22	Upper arm, 3 × 5 /5 min, 200 mmHg	Liver synthetic function
Teo [23]	2020	Singapore	24	26	64 ± 11.2	67 ± 8.4	20/4	19/7	Upper arm, 4 × 5 /5 min, 200 mmHg (for patients with a SBP ≥175 mmHg, the cuff was inflated to 25 mmHg above SBP)	ALT lever on POD 1
Wu [22]	2020	China	34	39	50.7 ± 9.9	49.7 ± 10.4	29/5	31/8	Right arm, 3 × 5 /5 min, 200 mmHg	ALT and AST levers on POD 1
Qi [21]	2021	China	102	106	30.2 ± 5.9	30.0 ± 5.7	37/65	38/68	Right arm, 3 × 5 /5 min, 200 mmHg	Postoperative peak levers of ALT and AST
Hardt [12]	2023	Germany	51	51	68.2 ± 12.2	62.4 ± 12.7	33/18	36/15	Upper arm, 3 × 5 /5 min, 200 mmHg or 50 mmHg above the patient’s SBP	ALT and AST levers on POD 1
Kong [19]	2023	China	25	27	54.48 ± 9.77	53.81 ± 12.13	16/9	17/10	Right arm, 3 × 5 /5 min, 26 kPa	Liver synthetic function
Gao [20]	2023	China	20	20	55.6 ± 4.6	57.8 ± 7.2	12/8	11/9	Right arm, 3 × 5 /5 min, 200 mmHg	Liver synthetic function

*RIPC* Remote ischemic preconditioning, *ALT* Alanine transaminase, *AST* Aspartate aminotransferase, *TBIL* Total bilirubin, *ICG* Indocyanine green, *SBP* Systolic blood pressure, *POD 1* postoperative day 1, *UK* United Kingdom

### Assessment of bias risk

**Fig. 2:**
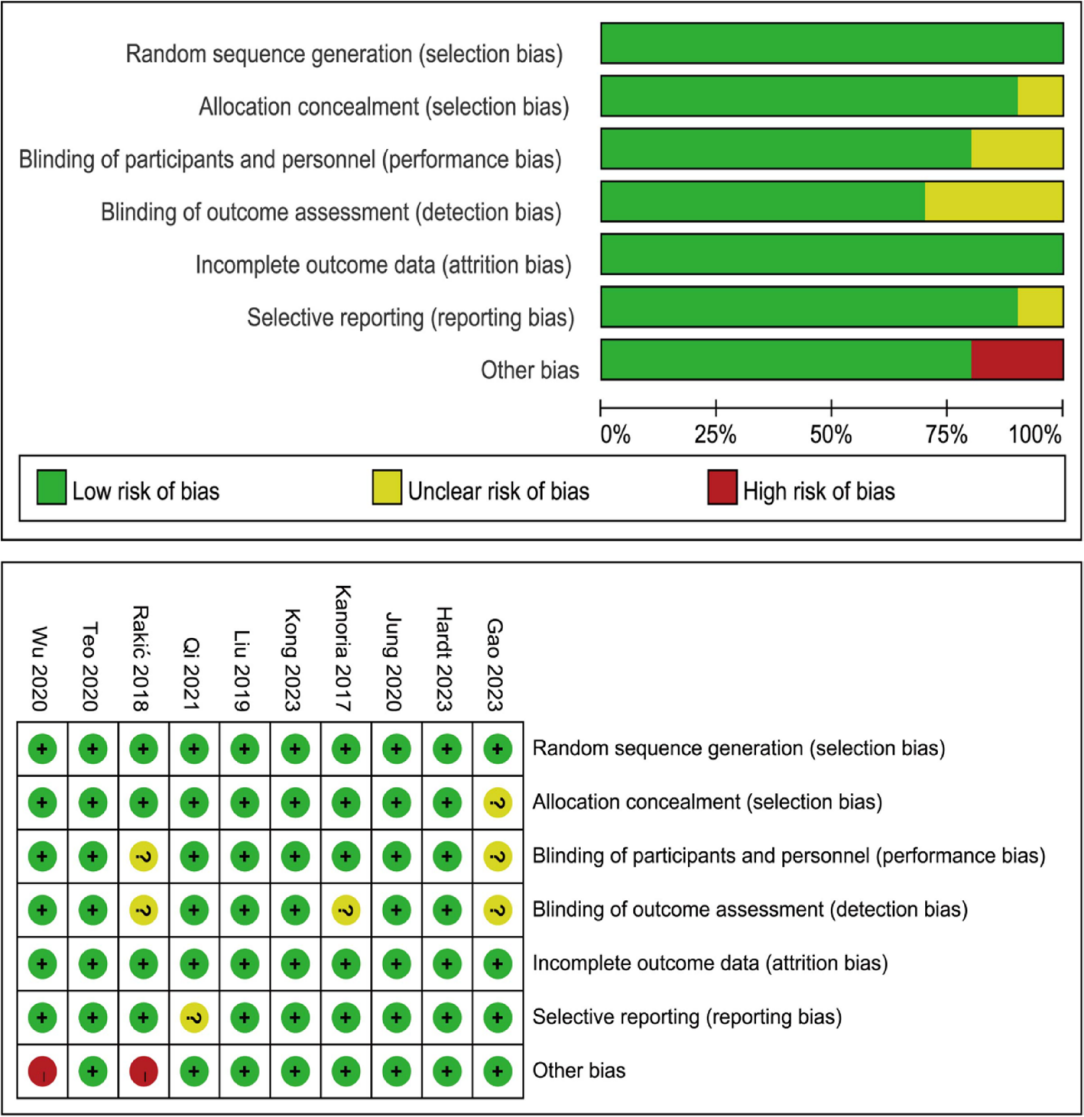
Risk of bias of the included studies

The Cochrane Collaborative Bias Risk tool was used to evaluate the risk of bias in the included RCTs, as shown in Fig. 2. Seven studies [12, 13, 19, 21, 23–25] were considered to have low risk bias in either six or seven measures, and no measure was considered high risk. One study [20] was considered to have low risk of bias in four measures, and the remaining three measures were considered to be uncertain. Only two studies [14, 22] were considered to have high risk of bias measures, with the remaining studies presenting a low risk.

### Effects of RIPC on primary outcomes

**Fig. 3:**
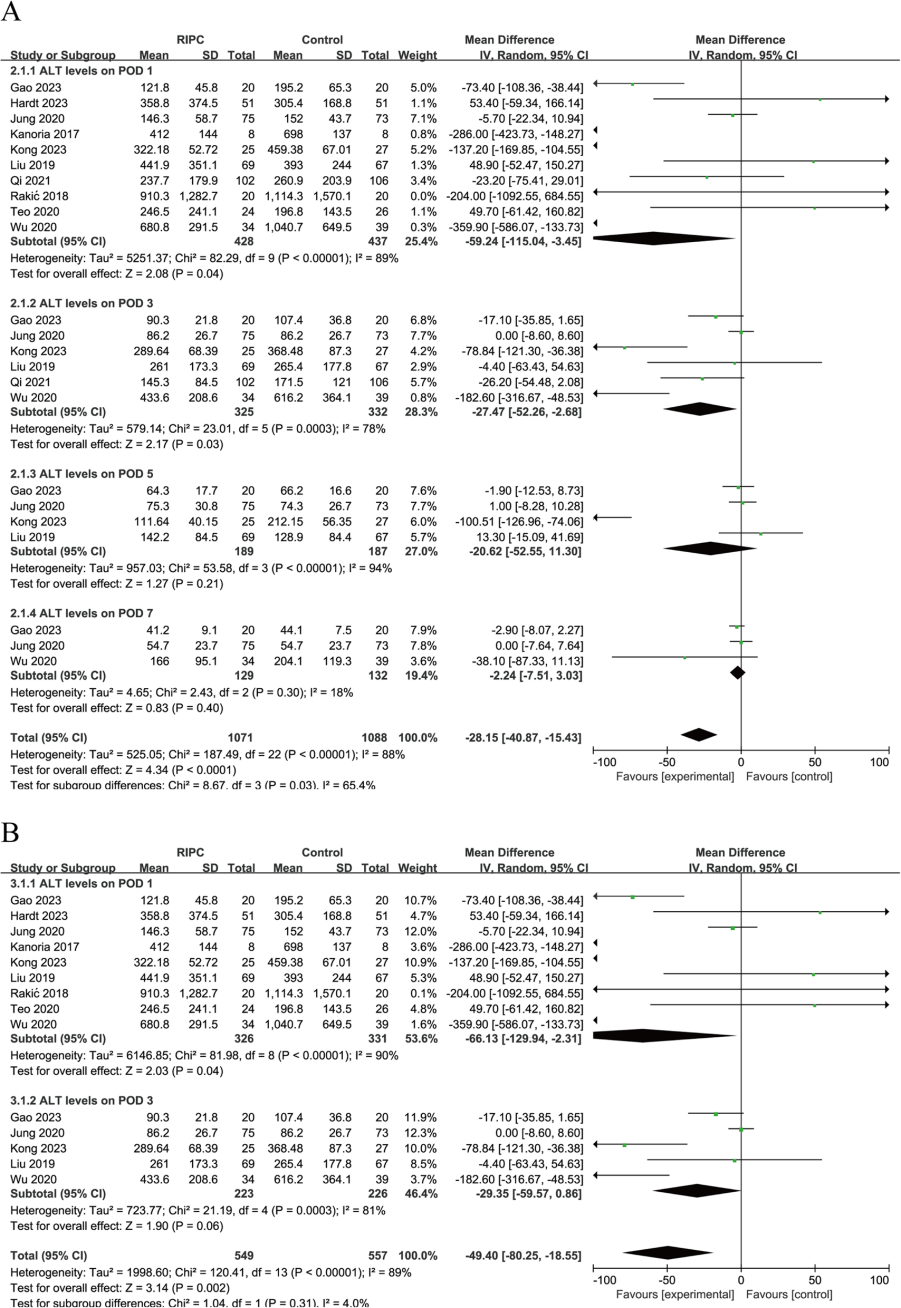
**A**, Forest plots for postoperative ALT levels. **B**, Forest plots of the sensitivity analysis

**Fig. 4:**
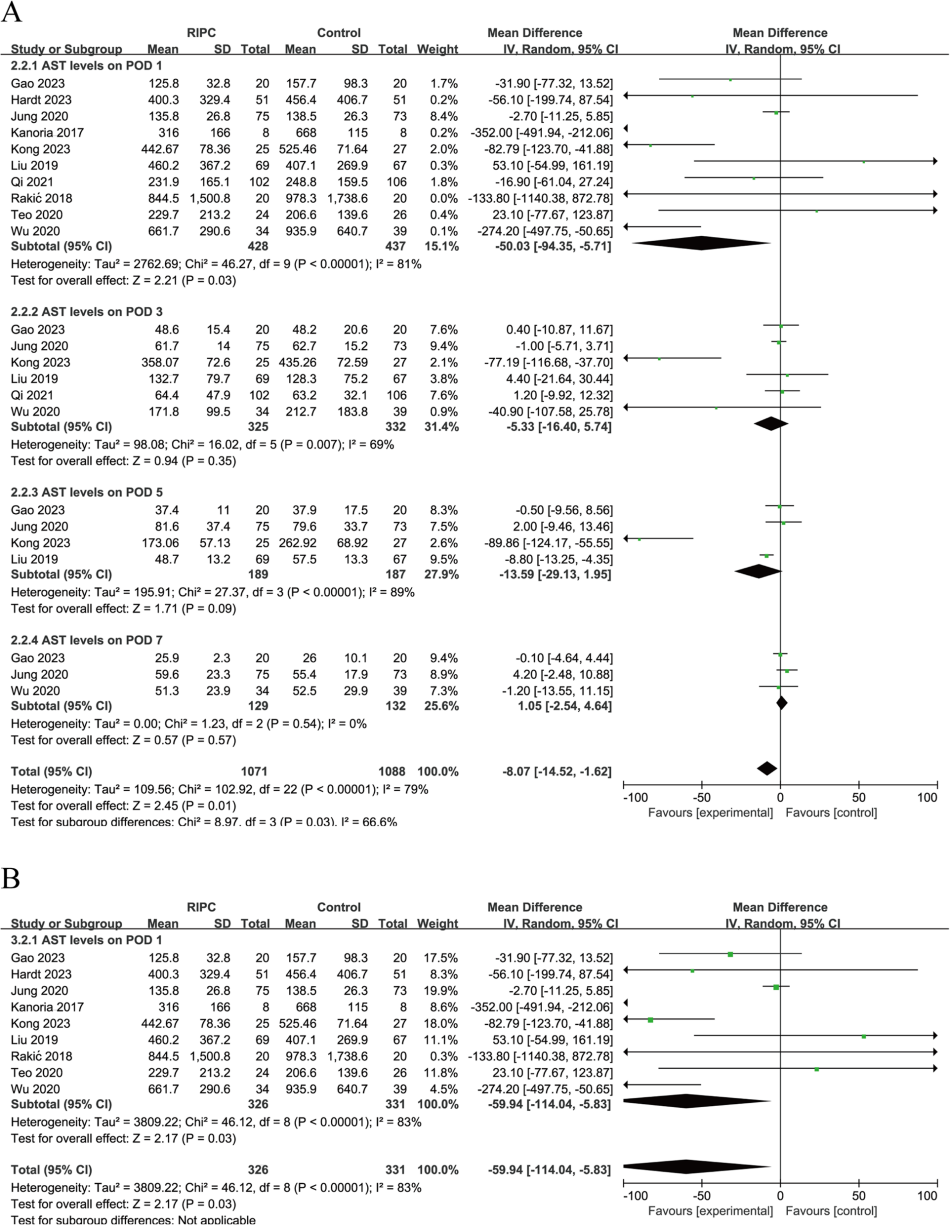
**A**, Forest plots for postoperative AST levels. **B**, Forest plots of the sensitivity analysis

All 10 studies [12–14, 19–25] evaluated ALT and AST levels on postoperative day (POD) 1, six [13, 19–22, 24] on POD 3, four on POD 5, and three on POD 7. Given that heterogeneity was high (*I*^*2*^ = 88%; *I*^*2*^ = 79%), the random-effects model was applied to pool the data. The results showed that ALT levels of the RIPC group were lower than the control group on POD 1 (WMD = − 59.24, 95% CI: − 115.04 to − 3.45; *P* = 0.04) and POD 3 (WMD = − 27.47, 95% CI: − 52.26 to − 2.68; *P* = 0.03) (Fig. 3A). AST levels of the RIPC group were lower than the control group on POD 1 (WMD = − 50.03, 95% CI: - 94.35 to − 5.71; *P* = 0.03) (Fig. 4A). Similar heterogeneities and pooled estimates of ALT and AST levels on POD 1 were obtained when one of the RCTs was excluded, suggesting that the results were stable, and that the evidence quality was considered moderate (Figs. 3B, and 4B). However, the pooled estimates of ALT levels obtained on POD 3 changed (WMD = − 29.35, 95% CI: − 59.57 to 0.86; *P* = 0.06) (Fig. 3B). This indicated that the analysis results of ALT levels on POD 3 were unstable.

Six RCTs [13, 19–22, 24] evaluated TBIL levels on POD 1 and POD 3, four on POD 5, and four on POD 7. Given that heterogeneity was high (*I*^*2*^ = 84%), the random-effects model was applied to pool the data. The results showed no significant differences in postoperative TBIL levels between the RIPC group and the control group (Supplementary Fig. S1). The robustness of these results was confirmed by a sensitivity analysis.

Three RCTs [21, 22, 24] evaluated ALB levels on POD 1 and POD 3. There was low heterogeneity among these RCTs (*I*^*2*^ = 0%), and therefore the fixed-effects model was applied to pool the data. The results showed no significant differences in postoperative ALB levels between the RIPC group and the control group (Supplementary Fig. S2). The robustness of these results was confirmed by a sensitivity analysis.

### Effects of RIPC on secondary outcomes

Nine RCTs [12, 13, 19–21, 23–25] provided data on operative times. RIPC interventions did not significantly alter the duration of surgeries (WMD = 1.54, 95% CI: - 4.09 to 7.16; *P* = 0.59) (Supplementary Fig. S3). In seven RCTs [12, 19–24], a total of 661 patients were compared and evaluated for intraoperative blood loss. RIPC did not reduce intraoperative bleeding in hepatectomy patients when compared to the control group (WMD = 0.40, 95% CI: - 9.20 to 10.01; *P* = 0.93) (Supplementary Fig. S4). Heterogeneities in the above analyses were low, and therefore the fixed-effects model was applied to pool the data. In addition, the stability of the above results was confirmed using a sensitivity analysis.

Five RCTs [12, 13, 19, 20, 24] provided data on postoperative hospital stays. The heterogeneity of the analysis was moderate (*I*^*2*^ = 56%), and therefore the random-effects model was applied to pool the data. RIPC interventions did not significantly alter postoperative hospital stays (SMD = − 0.53; 95% CI: − 1.28 to 0.22, *P* = 0.17) (Supplementary Fig. S5). The robustness of these results was confirmed by a sensitivity analysis.

Seven RCTs [12, 13, 20, 21, 23–25] provided data on postoperative complications. The heterogeneity of the analysis was moderate (*I*^*2*^ = 0%), and therefore the fixed-effects model was applied to pool the data. Based on the Modified Clavien Grading System, a meta-analysis showed no significant difference in postoperative complications between RIPC and control groups in grades I-II (SMD = 1.13; 95% CI: 0.77 to 1.67, *P* = 0.53) and grades III-V (SMD = 1.39; 95% CI: 0.68 to 2.82, *P* = 0.36) (Supplementary Fig. S6). There was also no statistical difference in intra-abdominal collection or bleeding, bile leakage after hepatobiliary and pancreatic surgery (PHBL), pulmonary complications, and wound infection or bleeding between the two groups. The robustness of these results was confirmed by a sensitivity analysis.

The other four outcomes could not be meta-analyzed due to data availability limitations, including intraoperative transfusion, ICG clearance, hepatocyte apoptosis index, and postoperative TNF-α levels. However, two studies [19, 20] with a total of 92 patients (45 in the RIPC group and 47 in the control group) evaluated postoperative TNF-α levels on POD 1, suggesting that RIPC interventions could inhibit inflammatory responses by reducing TNF-α in patients undergoing hepatectomies.

### Subgroup analysis

**Fig. 5:**
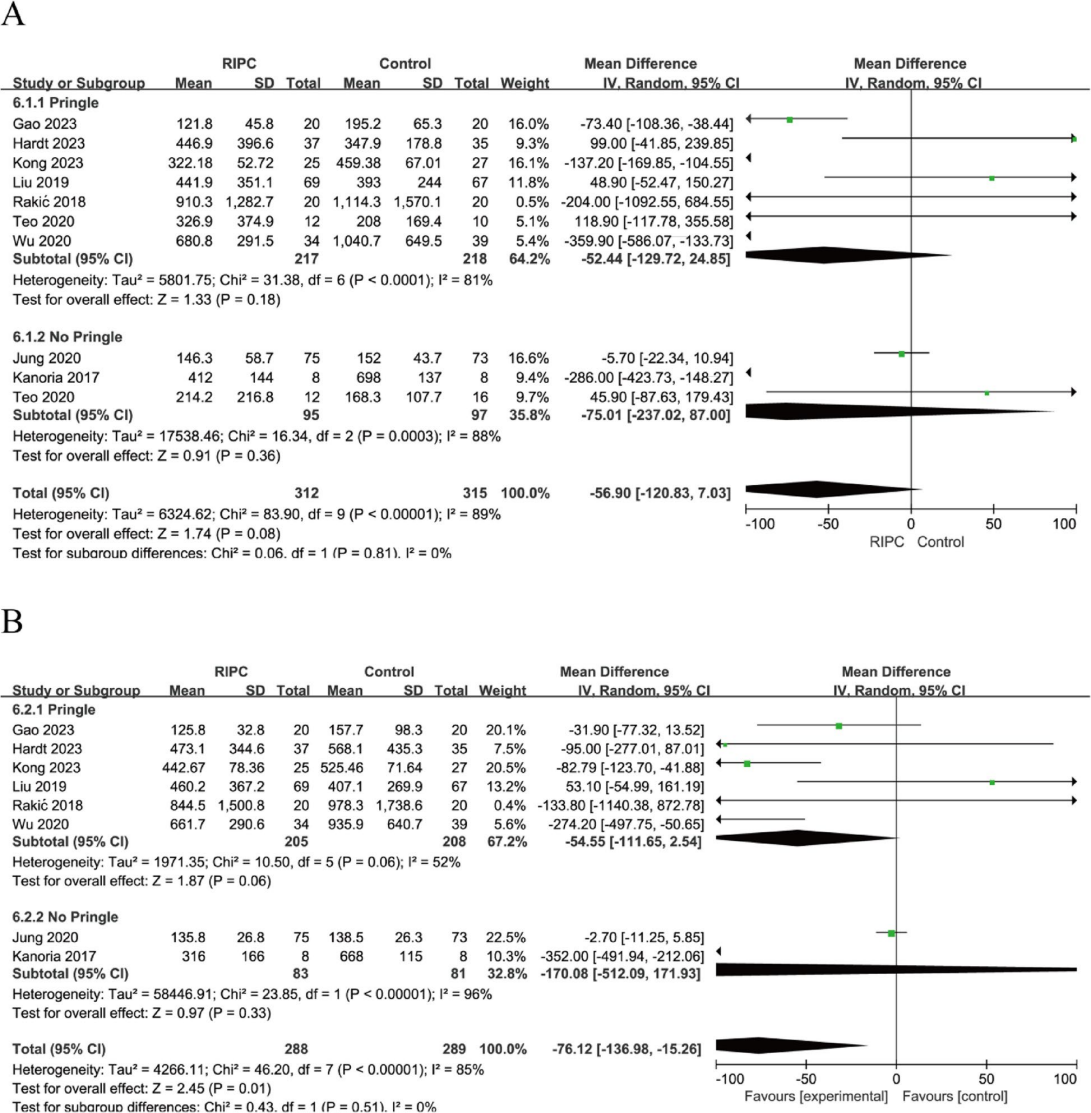
Forest plots of a subgroup analysis on the use of the Pringle maneuver. **A**, ALT levels on POD 1. **B**, AST levels on POD 1

**Fig. 6:**
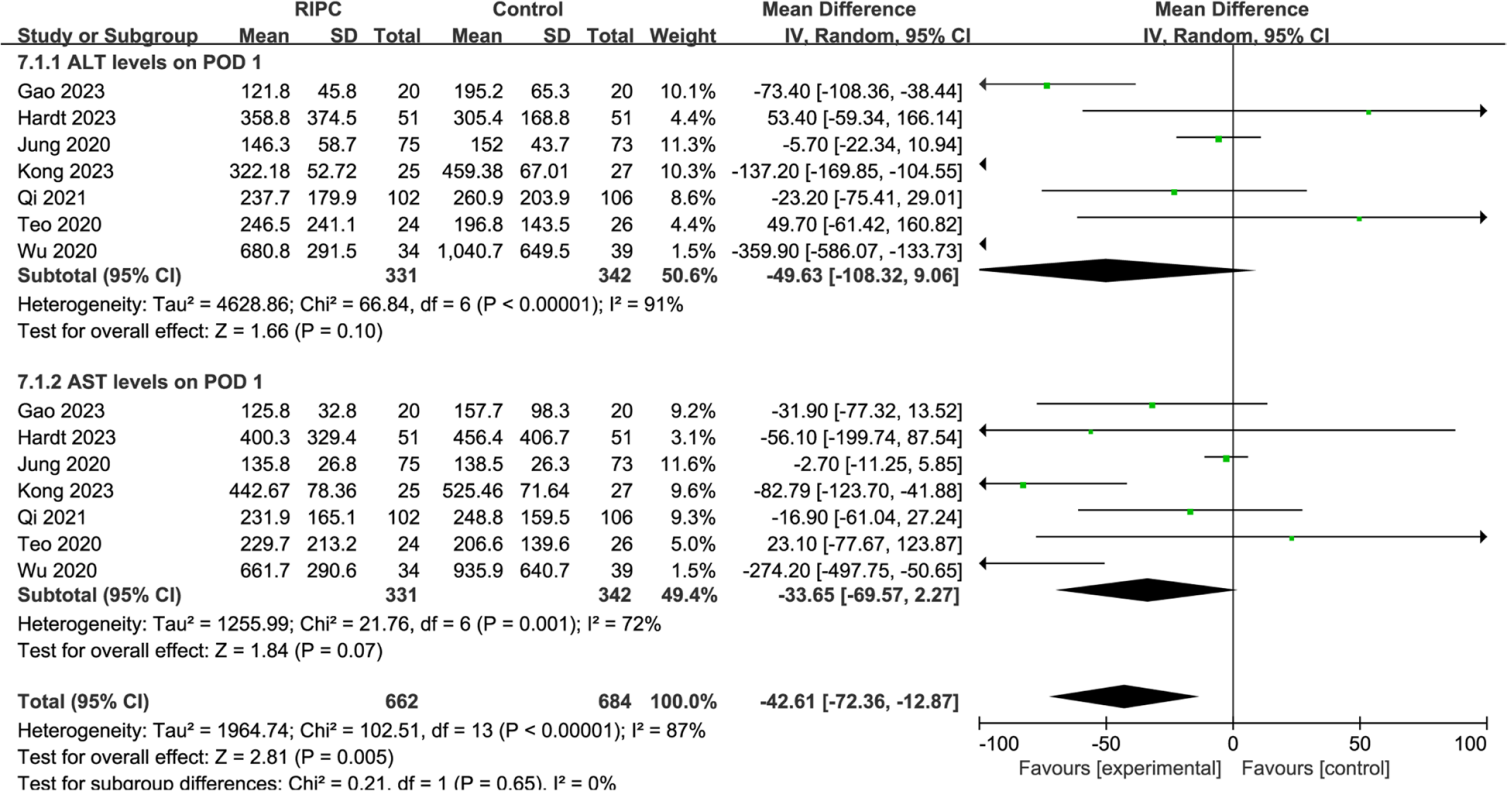
Forest plot of a subgroup analysis on the use of propofol for anesthesia

The use of the Pringle maneuver during hepatectomies is highly likely to cause HIRI [5]. Therefore, a subgroup analysis was performed to determine whether the Pringle maneuver should be used routinely. In addition, a subgroup analysis was also conducted to determine whether propofol was used as anaesthesia. The results showed that ALT and AST levels on POD 1 were not significantly different between the RIPC and control groups, regardless of whether the Pringle maneuver or propofol were used (induction only or both induction and maintenance) (all *P* > 0.05) (Figs. 5, and 6). The subgroup analysis did not significantly reduce heterogeneity.

### Publication bias

Supplementary Fig. S7 illustrates a funnel plot for the assessed postoperative ALT, AST, TBIL, and ALB levels. Funnel plots showed asymmetric patterns, suggesting a possible publication bias in this meta-analysis.

## Discussion

### Comparative analysis with other systematic reviews

The first systematic review [16] on this topic was published online in January 2021, and included seven studies with a total of 459 patients. The evaluation results showed that ALT and AST levels on POD 1 in the RIPC group were lower. However, it included a study by Gao and colleagues [26] which was RIpostC, and results changed significantly when this study was excluded. The researchers also noted that the studies included in the meta-analysis were highly heterogeneous, and that the results required cautious interpretation. Based on current evidence, the researchers suggested that RIPC did not alleviate HIRI in patients undergoing hepatectomy, which is inconsistent with our findings. The researchers also found that levels of ALT and AST on POD 1 were not significantly different, regardless of whether vascular control techniques (Pringle maneuver) were used, which is consistent with our findings. In addition, our meta-analysis showed that when propofol was used for anesthesia (induction only or both induction and maintenance), RIPC interventions did not reduce ALT or AST levels on POD 1.

The second systematic review [15] on this topic was published in an Epub format in December 2021. Six papers were included, with a total of 216 patients who underwent RIPC and 212 patients who served as controls. The reported ALT, AST and TBIL levels in the RIPC group were significantly lower than those in the control group on POD 1, suggesting that RIPC had a strong short-term hepatoprotective effect against HIRI, which corroborates the results found in our study. The researchers also found a weak hepatoprotective effect of RIPC in patients with cirrhosis due to their higher sensitivity to HIRI. This finding was not observed in our study. The researchers suggested that long-term effects of RIPC should be considered in future studies. However, the inclusion of a RCT including liver transplant recipients was a limiting factor for that study, which was excluded in our review.

### Clinical implications

The main objective of this systematic review was to evaluate the potential beneficial effect of RIPC in reducing HIRI in the residual livers of patients undergoing hepatectomy. It should be noted that, in order to be as broad and comprehensive as possible in terms of the number of publications and the outcomes evaluated, studies ranging from liver transplant donors to cirrhotic patients were all included in this review. Therefore, the results obtained here have universal applicability. In addition, each postoperative complication that was suitable for meta-analysis was evaluated individually, rather than as a mere ‘incidence’.

RIPC is the administration of multiple transient cycles of ischemia/reperfusion, usually at a remote site or organ far from the target organ [13]. The application of RIPC is generally well tolerated, does not cause substantial harm to the patient, and does not interfere with the surgical process. Therefore, its application in clinical practice is easy to perform and may bring potential benefits to patients.

During the qualitative analysis of the included studies, it was noted that several studies reported a decrease in liver transaminases on POD 1 in hepatectomy patients with RIPC [14, 19, 22, 25]. Two studies have also reported a decrease in TBIL levels on POD 5 and POD 7 [14, 19]. However, no differences between the RIPC and control groups were found in other studies, leading to the conclusion that RIPC is ineffective in reducing HIRI. However, it is worth noting that in the first published RCT, RIPC was implemented through three 10-min cycles of alternate ischemia and reperfusion to the leg [25], while in the rest of the subsequent studies, the ischemia and reperfusion time of RIPC were both 5-min cycles, which may account for these differences.

The qualitative analysis of the included studies showed that RIPC produces poor results in living donor hepatectomies [13, 21], and it appears that prolonged duration of surgeries also cause RIPC to gradually lose its protective effects [12, 13, 20, 23]. In addition, one study also analyzed a subgroup of patients with cirrhosis and indicated that the effect of RIPC on postoperative ALT levels in hepatectomy patients was not affected by cirrhosis [23]. Furthermore, two studies suggested that RIPC interventions can inhibit inflammatory responses by reducing TNF-α levels on POD 1 in hepatectomy patients, which warrants further investigation [19, 20].

In this meta-analysis, RIPC was found to reduce ALT and AST levels on POD 1 in patients undergoing hepatectomies. These outcomes are considered clinically relevant because ALT and AST levels are associated with liver synthetic function, suggesting that RIPC can alleviate early HIRI after hepatectomies. Similarly, although TBIL levels are considered a very sensitive indicator of liver failure after hepatectomy, there was no significant difference in postoperative TBIL levels between the RIPC and control groups. In a subgroup analysis, RIPC interventions were not found to reduce ALT or AST levels on POD 1 irrespective of the use of the Pringle maneuver or propofol.

On the other hand, there appeared to be negligible differences in clinical practice in terms of overall outcomes, such as intraoperative transfusion, length of hospital stays, and hepatocyte apoptosis index. In addition, conflicting results of previous studies on this topic have undoubtedly hindered the assessment of the effectiveness of RIPC interventions.

While most studies have standardized their protocols, forming a consensus of three 5-min cycles of alternate ischemia and reperfusion, other studies with contradictory results suggest that this may not be ideal [12–14, 19–24]. This may be due to the existence of two windows of protection in ischemic preconditioning [27, 28]. The first window of protection (also known as the classical protective window) occurs immediately after ischemic preconditioning, has a strong effect, and lasts for 2 to 3 h, which may be related to the release of endogenous substances (such as adenosine, bradykinin, and nitric oxide). The second window of protection occurs 12 to 24 h after ischemic preconditioning, has a weak effect, and lasts for 72 to 96 h, which may be related to the endogenous substances mediating cell signaling pathways and gene regulation.

### Limitations of this study

This study has some limitations. Firstly, the peak value of postoperative liver function index may better reflect the status of postoperative HIRI. Given that most researchers did not disclose all data associated with postoperative liver function indexes, peak levels could not be analysed. Secondly, there were great heterogeneities in the meta-analysis results of postoperative ALT and AST levels. However, sensitivity and subgroup analyses did not reduce the heterogeneity of the meta-analysis, and failed to explore sources of high heterogeneity. Thirdly, subgroup analyses of patients’ preoperative liver function status and age were not performed due to lack of relevant information, as the role of ischemic preconditioning may be diminished in patients with cirrhosis or in the elderly. Despite the above limitations, this study is still the most accurate meta-analysis performed to date.

## Conclusions

There were evident heterogeneities in several important outcomes in this study (including postoperative ALT, AST, and TBIL levels, and length of hospital stays), as well as differences found in qualitative evaluations. In conclusion, although RIPC does not cause harm to patients and has some short-term hepatoprotective effects on HIRI during hepatectomies, there is insufficient data to support its routine use in clinical practice to improve clinical outcomes. Therefore, additional RCTs with technical scientific rigor and standardization are needed.

### Supplementary Information

**Supplementary Material 1.**

## Data Availability

Raw data relevant to the conclusions of this study will be provided by the corresponding authors upon reasonable request.

## Abbreviations

RIPC: Remote ischemic preconditioning
RIpostC: Remote ischemic postconditioning
HIRI: Hepatic ischemia-reperfusion injury
RCTs: Randomized controlled trials
ALT: Alanine aminotransferase
AST: Aspartate transaminase
TBIL: Total bilirubin
ALB: Albumin
ICG: Indocyanine green
POD: Postoperative day
WMD: Weight mean difference
CI: Confidence interval
OR: Odds ratio
