# Supplementary material for: Effects of remote ischemic preconditioning in hepatectomy: a systematic review and meta-analysis
Source: BMC Anesthesiol. 2024 Mar 26;24:118. doi: 10.1186/s12871-024-02506-9 (PMC10964603; doi:10.1186/s12871-024-02506-9)
Supplement: Supplementary file 1 — Supplementary Material 1. [file 12871_2024_2506_MOESM1_ESM.pdf]

Supplementary Table S1 The search strategy.

Database	Search strategy
PubMed	((((((((Remote Ischemic Conditioning) OR (Limb Ischemic Conditioning)) OR (Remote ischemic treatment)) OR (Remote ischemic adaptation)) OR ((((((Remote ischemic preconditioning) OR (Distant ischemic preconditioning)) OR (ischemic preconditioning)) OR (Remote Ischemic Conditioning)) OR (remote ischemic adaptation)) OR (Limb ischemic preconditioning))) OR ((Remote ischemic perconditioning) OR (Limb ischemic perconditioning))) OR ((Remote ischemic postconditioning) OR (Limb ischemic postconditioning))) OR ((((((((RIC) OR (RIPerC)) OR (RIPostC)) OR (RIP)) OR (RIPC)) OR (RPC)) OR (IperC)) OR (rIPC))) AND (((((hepatic ischemia-reperfusion) OR (liver graft)) OR (liver transplantation)) OR (liver resection)) OR (hepatectomy))
OVID	(Remote Ischemic Conditioning or Limb Ischemic Conditioning or Remote ischemic treatment or Remote ischemic adaptation or (Remote ischemic preconditioning or Distant ischemic preconditioning or ischemic preconditioning or Remote Ischemic Conditioning or remote ischemic adaptation or Limb ischemic preconditioning) or (Remote ischemic perconditioning or Limb ischemic perconditioning) or (Remote ischemic postconditioning or Limb ischemic postconditioning) or (RIC or RIPerC or RIPostC or RIP or RIPC or RPC or IperC or rIPC)).mp. and (hepatic ischemia-reperfusion or liver graft or liver transplantation or liver resection or hepatectomy).m_titl. [mp=ti, ab, tx, ct, sh, bt, ot, nm, hw, fx, kf, ox, px, rx, an, ui, ds, on, sy, ux, mx]
Web of Science	(((((TS=(Remote Ischemic Conditioning or Limb Ischemic Conditioning or Remote ischemic treatment or Remote ischemic adaptation)) OR TS=(Remote ischemic preconditioning or Distant ischemic preconditioning or ischemic preconditioning or Remote Ischemic Conditioning or remote ischemic adaptation or Limb ischemic preconditioning)) OR TS=(Remote ischemic perconditioning or Limb ischemic perconditioning)) OR TS=(Remote ischemic postconditioning or Limb ischemic postconditioning)) OR TS=(RIC or RIPerC or RIPostC or RIP or RIPC or RPC or IperC or rIPC)) AND TS=(hepatic ischemia-reperfusion or liver graft or liver transplantation or liver resection or hepatectomy)
Cochrane library clinical trial databases	(Remote Ischemic Conditioning or Limb Ischemic Conditioning or Remote ischemic treatment or Remote ischemic adaptation or (Remote ischemic preconditioning or Distant ischemic preconditioning or ischemic preconditioning or Remote Ischemic Conditioning or remote ischemic adaptation or Limb ischemic preconditioning) or (Remote ischemic perconditioning or Limb ischemic perconditioning) or (Remote ischemic postconditioning or Limb ischemic postconditioning) or (RIC or RIPerC or RIPostC or RIP or RIPC or RPC or IperC or rIPC)) and (hepatic ischemia-reperfusion or liver graft or liver transplantation or liver resection or hepatectomy) in Title Abstract Keyword - (Word variations have been searched)
Embase	#1<br>'limb ischemic conditioning':ab,ti OR 'remote ischemic treatment':ab,ti OR 'remote ischemic preconditioning':ab,ti OR 'distant ischemic preconditioning':ab,ti OR 'ischemic preconditioning':ab,ti OR 'remote ischemic conditioning':ab,ti OR 'remote

ischemic adaptation':ab,ti OR 'limb ischemic preconditioning':ab,ti OR 'remote ischemic perconditioning':ab,ti OR 'limb ischemic perconditioning':ab,ti OR 'remote ischemic postconditioning':ab,ti OR 'limb ischemic postconditioning':ab,ti OR ric:ab,ti OR riperc:ab,ti OR ripostc:ab,ti OR rip:ab,ti OR rpc:ab,ti OR iperc:ab,ti OR ripc:ab,ti

#2

'hepatic ischemia-reperfusion':ab,ti OR 'liver graft':ab,ti OR 'liver transplantation':ab,ti OR 'liver resection':ab,ti OR hepatectomy:ab,ti

#3

#1 AND #2

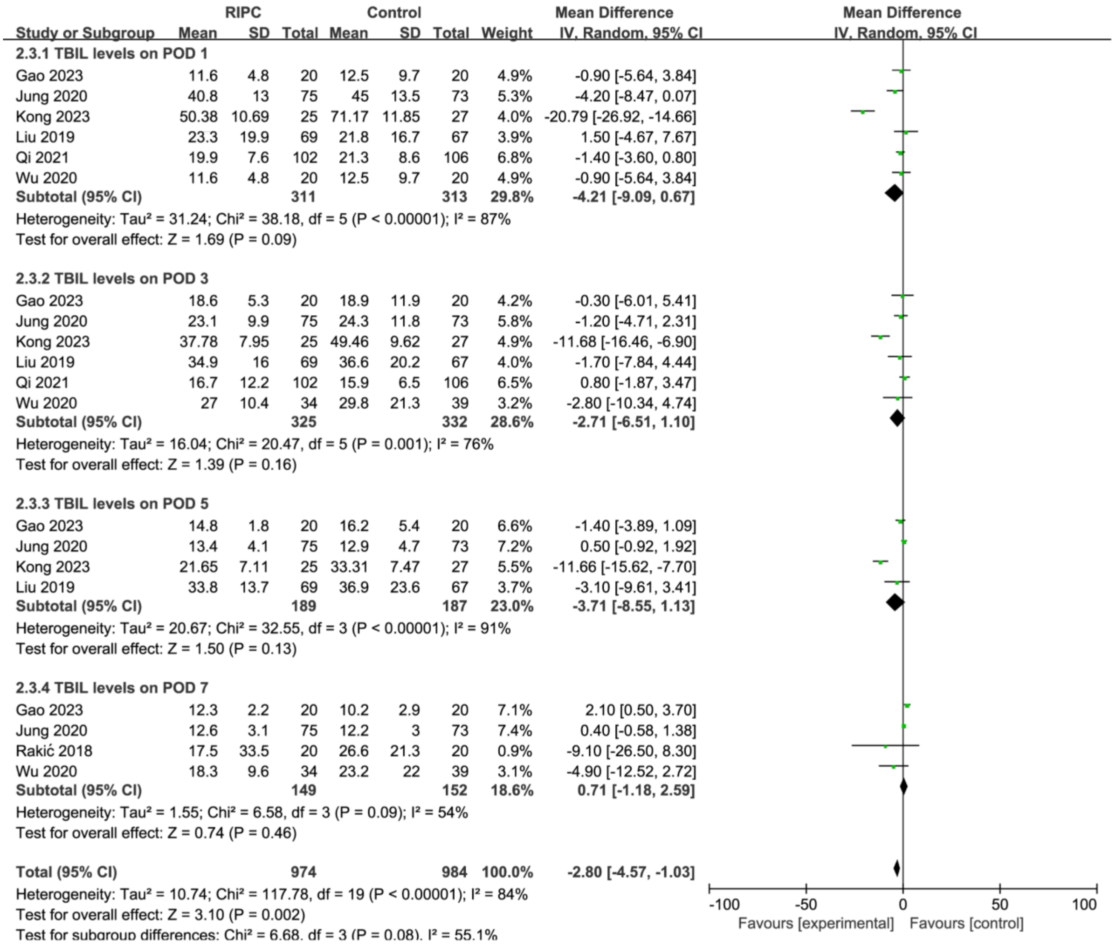

Supplementary Figure S1 Forest plot for postoperative TBIL levels.

Study or Subgroup	RIPC Mean	RIPC SD	RIPC Total	Control Mean	Control SD	Control Total	Weight	Mean Difference IV, Fixed, 95% CI
**2.4.1 ALB levels on POD 1**								
Liu 2019	34.3	3.6	69	33.6	3.8	67	11.9%	0.70 [-0.54, 1.94]
Qi 2021	38.1	3.4	102	37.8	2.7	106	26.4%	0.30 [-0.54, 1.14]
Wu 2020	35.6	3.6	34	36.6	3.9	39	6.2%	-1.00 [-2.72, 0.72]
**Subtotal (95% CI)**			**205**			**212**	**44.5%**	**0.23 [-0.42, 0.87]**
Heterogeneity: Chi² = 2.54, df = 2 (P = 0.28); I² = 21%								
Test for overall effect: Z = 0.69 (P = 0.49)								
**2.4.2 ALB levels on POD 3**								
Liu 2019	35.5	3.4	69	36.2	3.8	67	12.5%	-0.70 [-1.91, 0.51]
Qi 2021	39.1	2.6	102	39.3	2.6	106	37.0%	-0.20 [-0.91, 0.51]
Wu 2020	34	4.1	34	34.2	3.5	39	5.9%	-0.20 [-1.96, 1.56]
**Subtotal (95% CI)**			**205**			**212**	**55.5%**	**-0.31 [-0.89, 0.26]**
Heterogeneity: Chi² = 0.50, df = 2 (P = 0.78); I² = 0%								
Test for overall effect: Z = 1.06 (P = 0.29)								
**Total (95% CI)**			**410**			**424**	**100.0%**	**-0.07 [-0.50, 0.36]**
Heterogeneity: Chi² = 4.53, df = 5 (P = 0.48); I² = 0%								
Test for overall effect: Z = 0.33 (P = 0.74)								
Test for subgroup differences: Chi² = 1.49, df = 1 (P = 0.22), I² = 32.8%								

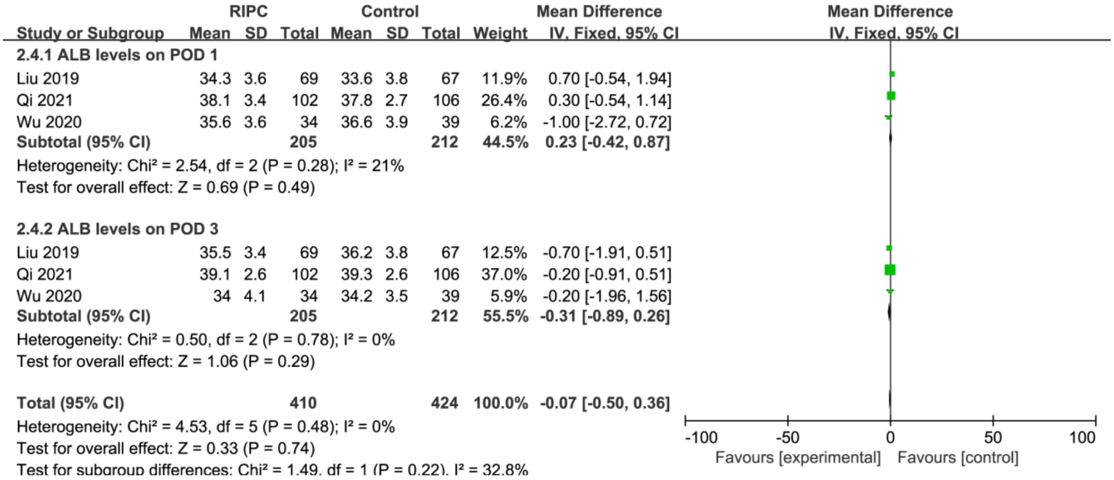

Supplementary Figure S2 Forest plot for postoperative ALB levels.

Study or Subgroup	RIPC Mean	RIPC SD	RIPC Total	Control Mean	Control SD	Control Total	Weight	Mean Difference IV, Fixed, 95% CI
Gao 2023	324.1	230.4	20	297.8	163.6	20	0.2%	26.30 [-97.54, 150.14]
Hardt 2023	257.5	129.3	51	269.8	162.4	51	1.0%	-12.30 [-69.27, 44.67]
Jung 2020	424.5	58.6	75	417.6	50.7	73	10.2%	6.90 [-10.74, 24.54]
Kanoria 2017	369	60.6	8	375	73.2	8	0.7%	-6.00 [-71.85, 59.85]
Kong 2023	146.16	26.25	25	146.93	25.56	27	15.9%	-0.77 [-14.87, 13.33]
Liu 2019	149.3	29.6	69	149.3	42.7	67	20.6%	0.00 [-12.38, 12.38]
Qi 2021	161.1	39.8	102	159	39.6	106	27.1%	2.10 [-8.69, 12.89]
Teo 2020	233	121.2	24	178	82.1	26	0.9%	55.00 [-2.85, 112.85]
Wu 2020	100.6	24.4	34	100.7	26.3	39	23.3%	-0.10 [-11.74, 11.54]
**Total (95% CI)**			**408**			**417**	**100.0%**	**1.54 [-4.09, 7.16]**
Heterogeneity: Chi² = 4.31, df = 8 (P = 0.83); I² = 0%								
Test for overall effect: Z = 0.54 (P = 0.59)								

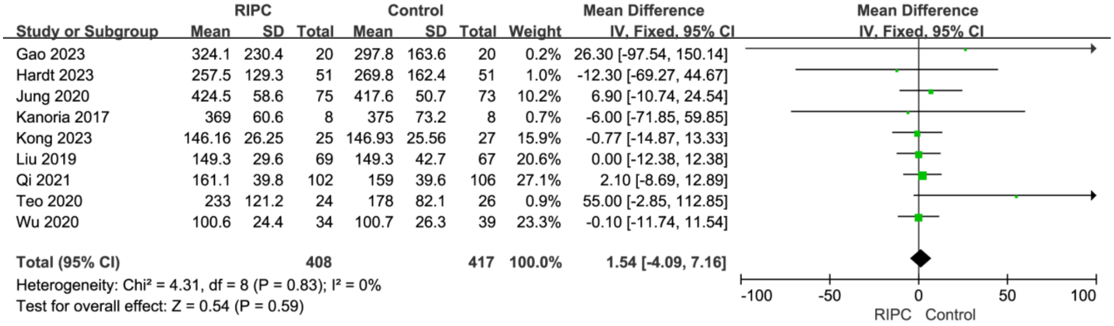

Supplementary Figure S3 Forest plot for operative times.

Study or Subgroup	RIPC Mean	RIPC SD	RIPC Total	Control Mean	Control SD	Control Total	Weight	Mean Difference IV, Fixed, 95% CI
Gao 2023	285.5	37.5	20	253	48.8	20	12.7%	32.50 [5.53, 59.47]
Hardt 2023	985.2	1,421.6	51	1,189.5	1,543.8	51	0.0%	-204.30 [-780.27, 371.67]
Kong 2023	541.2	80.74	25	546.67	89.05	27	4.3%	-5.47 [-51.62, 40.68]
Liu 2019	223.9	205.1	69	259.7	251.8	67	1.5%	-35.80 [-113.11, 41.51]
Qi 2021	62.4	34.8	102	65.1	43.9	106	79.9%	-2.70 [-13.44, 8.04]
Teo 2020	417	444.7	24	582	581.7	26	0.1%	-165.00 [-450.74, 120.74]
Wu 2020	237.9	168.2	34	274.4	180.4	39	1.4%	-36.50 [-116.51, 43.51]
**Total (95% CI)**			**325**			**336**	**100.0%**	**0.40 [-9.20, 10.01]**
Heterogeneity: Chi² = 9.25, df = 6 (P = 0.16); I² = 35%								
Test for overall effect: Z = 0.08 (P = 0.93)								

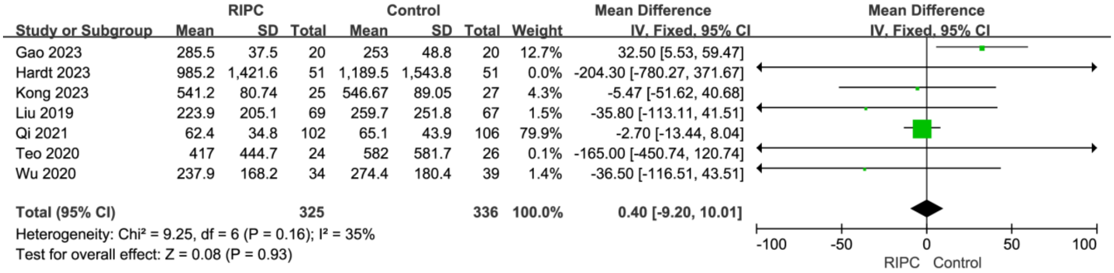

Supplementary Figure S4 Forest plot for intraoperative blood loss.

Study or Subgroup	RIPC Mean	RIPC SD	RIPC Total	Control Mean	Control SD	Control Total	Weight	Mean Difference IV, Random, 95% CI
Gao 2023	10.3	1.9	20	10.4	2.1	20	19.5%	-0.10 [-1.34, 1.14]
Hardt 2023	9.6	11.1	51	9.4	8.9	51	3.4%	0.20 [-3.70, 4.10]
Jung 2020	10.4	0.8	75	10.4	0.8	73	39.4%	0.00 [-0.26, 0.26]
Kong 2023	10.4	2.24	25	12.41	3.15	27	15.9%	-2.01 [-3.49, -0.53]
Liu 2019	6.6	1.5	69	7.5	4.4	67	21.7%	-0.90 [-2.01, 0.21]
**Total (95% CI)**			**240**			**238**	**100.0%**	**-0.53 [-1.28, 0.22]**
Heterogeneity: Tau² = 0.36; Chi² = 9.01, df = 4 (P = 0.06); I² = 56%								
Test for overall effect: Z = 1.38 (P = 0.17)								

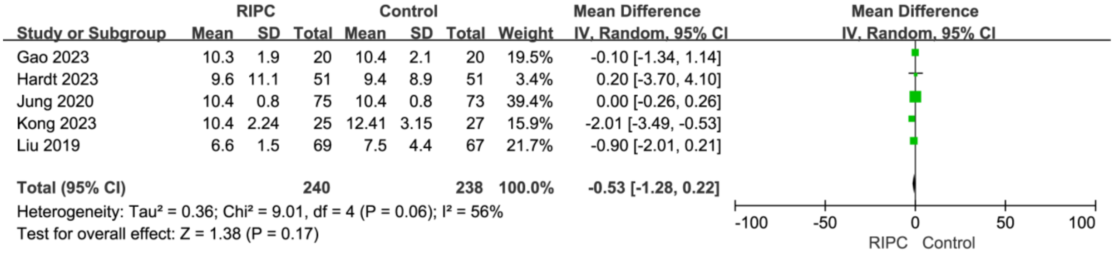

Supplementary Figure S5 Forest plot for postoperative hospital stays.

Study or Subgroup	RIPC Events	RIPC Total	Control Events	Control Total	Weight	Odds Ratio M-H, Fixed, 95% CI
**3.1.1 Grade I ~ II**						
Hardt 2023	18	51	13	51	10.2%	1.59 [0.68, 3.74]
Jung 2020	4	75	4	73	4.6%	0.97 [0.23, 4.04]
Liu 2019	24	69	26	67	20.8%	0.84 [0.42, 1.69]
Qi 2021	22	102	21	106	19.5%	1.11 [0.57, 2.18]
Teo 2020	5	24	3	26	2.8%	2.02 [0.43, 9.55]
**Subtotal (95% CI)**		**321**		**323**	**57.9%**	**1.13 [0.77, 1.67]**
Total events	73		67			
Heterogeneity: Chi² = 1.89, df = 4 (P = 0.76); I² = 0%						
Test for overall effect: Z = 0.62 (P = 0.53)						
**3.1.2 Grade III ~ V**						
Hardt 2023	17	51	14	51	11.3%	1.32 [0.57, 3.08]
Jung 2020	3	75	1	73	1.2%	3.00 [0.30, 29.52]
Liu 2019	1	69	1	67	1.2%	0.97 [0.06, 15.84]
Teo 2020	2	24	2	26	2.1%	1.09 [0.14, 8.42]
**Subtotal (95% CI)**		**219**		**217**	**15.8%**	**1.39 [0.68, 2.82]**
Total events	23		18			
Heterogeneity: Chi² = 0.57, df = 3 (P = 0.90); I² = 0%						
Test for overall effect: Z = 0.91 (P = 0.36)						
**3.1.3 Intra-abdominal collection or bleeding**						
Gao 2023	1	20	0	20	0.6%	3.15 [0.12, 82.16]
Jung 2020	0	75	1	73	1.8%	0.32 [0.01, 7.99]
Kanoria 2017	1	8	1	8	1.1%	1.00 [0.05, 19.36]
Liu 2019	0	69	1	67	1.8%	0.32 [0.01, 7.97]
**Subtotal (95% CI)**		**172**		**168**	**5.3%**	**0.76 [0.18, 3.17]**
Total events	2		3			
Heterogeneity: Chi² = 1.32, df = 3 (P = 0.72); I² = 0%						
Test for overall effect: Z = 0.38 (P = 0.70)						
**3.1.4 PHBL**						
Hardt 2023	5	51	8	51	8.7%	0.58 [0.18, 1.92]
Jung 2020	3	75	1	73	1.2%	3.00 [0.30, 29.52]
Liu 2019	1	69	0	67	0.6%	2.96 [0.12, 73.86]
**Subtotal (95% CI)**		**195**		**191**	**10.5%**	**0.99 [0.39, 2.54]**
Total events	9		9			
Heterogeneity: Chi² = 2.10, df = 2 (P = 0.35); I² = 5%						
Test for overall effect: Z = 0.02 (P = 0.98)						
**3.1.5 Pulmonary complications**						
Gao 2023	0	20	1	20	1.8%	0.32 [0.01, 8.26]
Kanoria 2017	3	8	4	8	3.0%	0.60 [0.08, 4.40]
Liu 2019	3	69	2	67	2.3%	1.48 [0.24, 9.13]
**Subtotal (95% CI)**		**97**		**95**	**7.1%**	**0.82 [0.25, 2.71]**
Total events	6		7			
Heterogeneity: Chi² = 0.82, df = 2 (P = 0.66); I² = 0%						
Test for overall effect: Z = 0.33 (P = 0.74)						
**3.1.6 Wound infections or oozing**						
Jung 2020	2	75	0	73	0.6%	5.00 [0.24, 105.95]
Kanoria 2017	1	8	2	8	2.1%	0.43 [0.03, 5.98]
Liu 2019	1	69	0	67	0.6%	2.96 [0.12, 73.86]
**Subtotal (95% CI)**		**152**		**148**	**3.3%**	**1.71 [0.38, 7.65]**
Total events	4		2			
Heterogeneity: Chi² = 1.64, df = 2 (P = 0.44); I² = 0%						
Test for overall effect: Z = 0.70 (P = 0.49)						
**Total (95% CI)**		**1156**		**1142**	**100.0%**	**1.13 [0.84, 1.52]**
Total events	117		106			
Heterogeneity: Chi² = 9.36, df = 21 (P = 0.99); I² = 0%						
Test for overall effect: Z = 0.84 (P = 0.40)						
Test for subgroup differences: Chi² = 1.27, df = 5 (P = 0.94), I² = 0%						

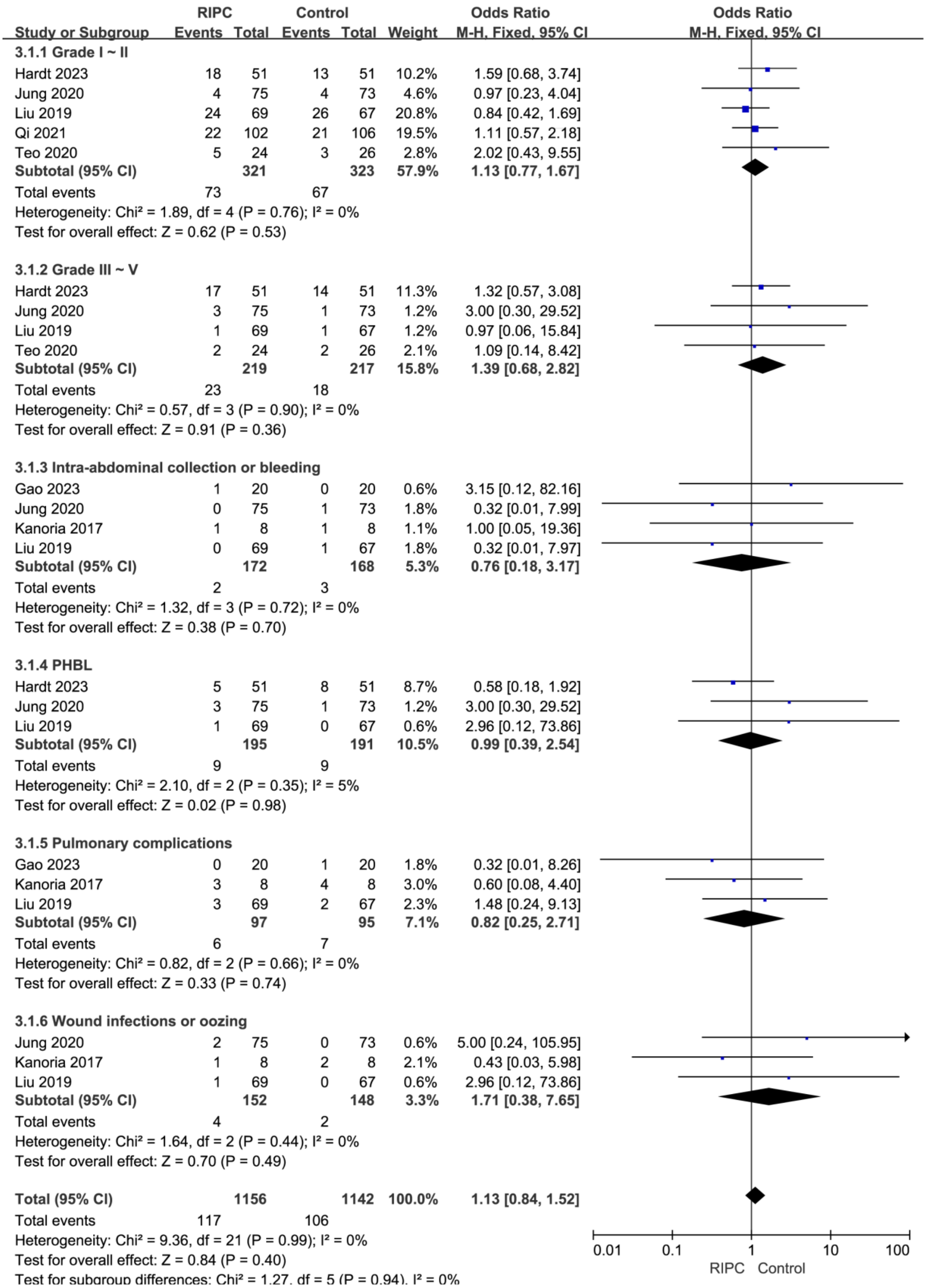

Supplementary Figure S6 Forest plot for postoperative complications. PHBL, bile leakage after hepatobiliary and pancreatic surgery.

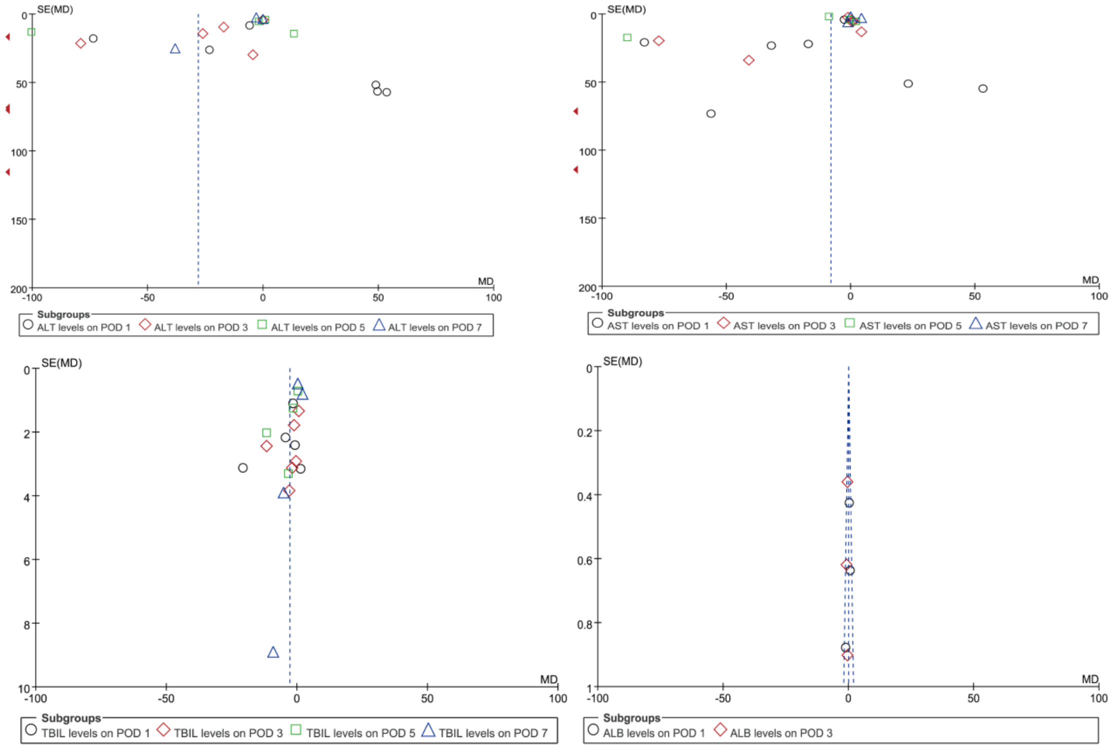

Supplementary Figure S7 Funnel plots based on postoperative primary outcomes.
